# Randomised controlled trial to evaluate the effectiveness of using the RD-1-based C-Tb skin test as a replacement for blood-based interferon-γ release assay for detection of, and initiation of preventive treatment for, tuberculosis infection: RID-TB:Dx study protocol

**DOI:** 10.1136/bmjopen-2021-050595

**Published:** 2021-12-30

**Authors:** Molebogeng X Rangaka, Yohhei Hamada, Trinh Duong, Henry Bern, Joanna Calvert, Marie Francis, Amy Louise Clarke, Alex Ghanouni, Vanessa Hack, Ellen Owen-Powell, Julian Surey, Karen Sanders, Helen L Booth, Angela Crook, Chris Griffiths, Robert Horne, Heinke Kunst, Marc Lipman, Mike Mandelbaum, Peter J White, Penny Whiting, Dominik Zenner, Ibrahim Abubakar

**Affiliations:** 1 Institute for Global Health, University College London, London, UK; 2 School of Public Health, and Clinical Infectious Disease Research Institute-AFRICA, University of Cape Town, Cape Town, South Africa; 3 MRC Clinical Trials Unit at UCL, Institute of Clinical Trials and Methodology, London, UK; 4 Centre for Behavioural Medicine, UCL School of Pharmacy, London, UK; 5 University College London Hospitals NHS Foundation Trust, London, UK; 6 Blizard Institute, Barts and The London School of Medicine and Dentistry, Queen Mary University of London, London, UK; 7 UCL Respiratory, Division of Medicine, University College London, London, UK; 8 Royal Free London Hospital NHS Foundation Trust, London, UK; 9 TB Alert, London, UK; 10 MRC Centre for Global Infectious Disease Analysis, Imperial College London, London, UK; 11 Modelling and Economics Unit, National Infection Service, Public Health England, London, UK; 12 Population Health Sciences, Bristol Medical School, University of Bristol, Bristol, UK; 13 Institute of Population Health Sciences, Queen Mary University of London, London, UK

**Keywords:** tuberculosis, clinical trials, diagnostic microbiology, preventive medicine

## Abstract

**Introduction:**

The predictive utility for incident tuberculosis (TB) of the purified protein derivative tuberculin skin test and region of difference 1 (RD1)-based interferon-gamma release assays (IGRA) is comparable; and either is recommended to test for latent TB infection (LTBI). Despite associated high costs of IGRA, sites participating in LTBI screening in many high-income settings pragmatically favour IGRA due to its higher specificity and simpler logistics. A new RD1-based skin test, C-Tb, could offer an acceptable and as accurate, cheaper alternative to IGRA. Evaluating the impact of C-Tb on process and patient-related outcomes would provide important information to help guide its use in LTBI testing strategies.

**Methods and analysis:**

This is a pragmatic multicentre, open-label, non-inferiority, randomised controlled trial. The trial will assess the initiation of LTBI treatment following a positive result of the randomised test as the primary outcome. Participants will be randomised to receive the C-Tb test (intervention) or IGRA (usual care, control) for initiation of treatment. We will enrol 1530 participants in England aged≥16 years who are eligible for LTBI testing and treatment according to UK guidance. In the C-Tb arm, skin induration will be assessed 2–3 days after intradermal injection and measured in millimetres of induration. Results of IGRA will be obtained in line with standard practice. Behavioural studies will explore people’s experiences, perspectives and preferences of LTBI testing and treatment. Economic analysis will estimate cost-effectiveness of changes to the diagnostic algorithm for LTBI. The protocol was developed with Patient and Public Involvement (PPI), which will continue throughout the trial.

**Ethics and dissemination:**

Ethics approval has been obtained from The NHS Health Research Authority (269485). We will share results of the trial in peer-reviewed journals and conferences.

**Trial registration number:**

EudraCT 2019-002592-34; ISRCTN17936038.

**Strengths and limitations of this study**

- This is the first trial to assess the impact of a novel C-Tb test on initiation of treatment for latent tuberculosis infection (LTBI) and process outcomes along the LTBI care cascade.
- Substudies will assess people’s experiences, perspectives and preferences of LTBI testing and treatment and estimate cost and cost-effectiveness of C-Tb, which provide additional evidence to guide testing algorithms.
- The trial will be conducted in England, largely in migrant populations and contacts of TB patients, and thus generalisability to other settings and populations may be limited.
- This is an open-label trial; knowing the allocation group might influence how clinicians manage participants offered preventive treatment.
- Initiation of LTBI treatment for the primary outcome will be based on prescription records.

## Introduction

Following major declines in tuberculosis (TB) incidence over most of the 20th century, there was a resurgence of the disease in the UK from the late 1980s to 2005,^1^ which then stabilised at relatively high levels but has been declining since 2011.^2^ In 2019, there were 4725 cases notified in England; an incidence of 8.4 cases per 100 000 population, still higher than most other Western European countries and three times as high as in the USA.^2^ TB disproportionately affects underprivileged communities, such as migrants and homeless people, with a higher incidence of disease and poorer outcomes associated with disadvantage. Addressing TB in these populations is critical for UK to achieve TB elimination.

The diagnosis and treatment of people diagnosed with latent TB infection (LTBI) can reduce the incidence of TB disease by halting progression of LTBI to TB disease, and consequently disrupting transmission in the community.^3^ LTBI screening and treatment for high risk groups such as new migrants from high TB incidence countries is thus recognised as an essential strategy to halt transmission in England.^4^

Currently available LTBI tests in England include the interferon-gamma release assays (IGRA) and the purified protein derivative tuberculin skin test (TST).^5^ The utility of the TST is limited by its poor specificity and sensitivity; and operational challenges.^6^ Its sensitivity and specificity were estimated to be 70% and 68%, respectively, in immunocompetent adults in a systematic review.^7^ IGRA while similar sensitivity for LTBI ranges 60%–80%, in contrast has specificity 90%–99%, depending on the population.^6 7^ Regardless of the differences in accuracy, head-to-head analyses in systematic reviews have shown the predictive performance for subsequent progression to active TB of the two tests to be comparable.^8 9^ Based on low-quality evidence, the WHO recommended that either test could be used for the diagnosis of LTBI in high-income low-incidence countries.^8 10^ The LTBI screening programme in the UK pragmatically offers IGRA to new migrants and other groups at high risk for TB infection and disease due to its higher specificity and relatively simpler logistics. The use of IGRA may lead to a smaller number of patients requiring treatment compared with TST without an increase in TB incidence in people who are not treated.^11^ Furthermore, IGRA does not require a return visit for evaluation of results within a specific time frame for results to remain valid, unlike TST. However, individuals who test positive on IGRA still currently need to return to care for TB investigations and initiation of preventive treatment. The use of IGRA as a primary test for TB infection follows the guidance in the 2016 National Institute for Health and Care Excellence guidelines for managing LTBI.^5^ Despite its apparent operational simplicity, blood-based detection of LTBI by IGRA is less cost-effective than TST.^12^ Moreover, although IGRA results could, in theory, be available on the same day, to offset costs, tests are run in batches every 5–14 days depending on local arrangements. This can result in delays in obtaining actionable results and eventual prescription of preventive treatment to eligible individuals, which offsets perceived advantages.

Statens Serum Institut has developed a new skin test (C-Tb), containing the recombinant antigens ESAT-6 (dimer) and CFP10 (monomer) derived from *Mycobacterium tuberculosis*. Similar to IGRA, and in contrast to the TST, C-Tb appears unaffected by previous BCG vaccination^13^ and HIV infection,^14^ both of which affect test performance of TST. Studies reported high overall concordance (94%), and similar sensitivity (74%) and specificity (96%) to the QuantiFERON-TB (QFT) Gold In Tube blood IGRA test.^15 16^ C-Tb could thus be an immunological improvement on the standard TST, and may therefore offer a cheaper, accurate and acceptable replacement or alternative to IGRA.^5^ However, there is no evidence of the impact of C-Tb use on patient and process outcomes along the LTBI care pathway from initiation to completion of treatment, or utility when used in current testing algorithms. In a previous study, participants at high risk for TB in an urban area in the US preferred initiation LTBI treatment based on TST results to those of the QFT Gold.^17^ While accuracy data alone are often used to register new diagnostic tools, any decision to replace a current tool with a new one should additionally consider the impact on patient-important outcomes evaluated in randomised studies.^18^

### Objectives

The overall aim of the RID-TB:DX trial is to evaluate whether C-Tb can be used as an alternative test to the interferon-gamma release assay in the screening of LTBI in England. The primary objective is to determine whether the proportion of participants initiating LTBI treatment based on C-Tb testing is at least as high as that based on the standard-of-care testing. The secondary objectives include: (1) to determine the safety of C-Tb test; (2) to determine the impact of C-Tb test on outcomes along the LTBI care pathway; (3) to evaluate the concordance and diagnostic accuracy of the C-Tb test compared with IGRA; (4) to assess modifiable behavioural factors influencing patient and provider engagement with LTBI testing (includes acceptability and other patient-important outcomes) and (5) to evaluate the cost-effectiveness and budget impact of combinations of new technologies to improve LTBI outcomes.

## Method and analysis

### Trial design

**Figure 1:**
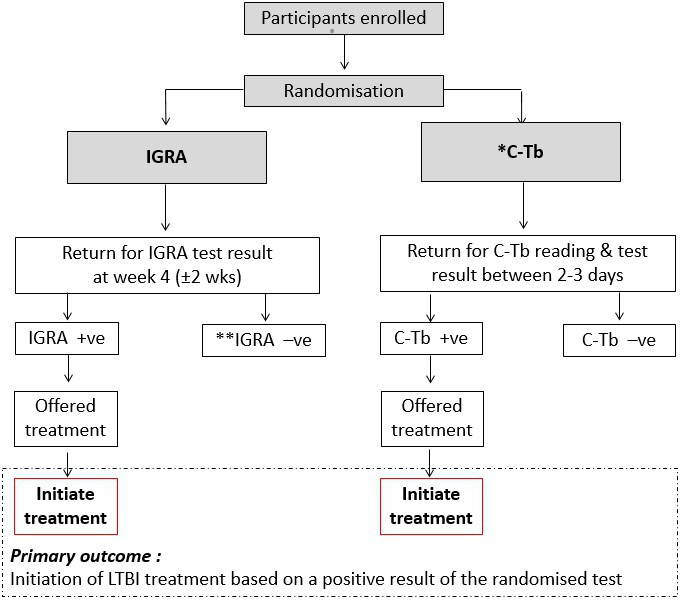
Trial schema. *IGRA test will also be done, as per usual care, with those testing C-Tb-negative but IGRA-positive being offered LTBI treatment; such patients who initiate treatment will not count as having achieved the primary endpoint. C-Tb assessors will be blinded to IGRA results. **On review on follow-up, test-negative individuals will be informed of their result and counselled on its interpretation. IGRA, interferon-gamma release assays; LTBI, latent tuberculosis infection.

A multicentre open-label non-inferiority randomised controlled trial with two parallel groups, C-Tb testing versus standard-of-care testing (IGRA), with 1:1 allocation ratio (figure 1).

### Study setting

The trial will recruit from primary and secondary care sites that implement systematic LTBI testing and treatment in England, UK. RID-TB: Diagnostics (Dx) is part of a 5-year programme of work (RID-TB) which is funded by the National Institute for Health Research (NIHR) (RP-PG-0217-20009 https://dev.fundingawards.nihr.ac.uk/award/RP-PG-0217-20009).

### Study population

The trial will enrol populations for which LTBI testing and treatment is recommended according to national guidance.^19^ The trial will largely recruit the priority risk groups for LTBI testing comprising individuals who are contacts of persons diagnosed with active TB and persons screened within the migrant screening programme.^19 20^ The LTBI migrant screening programme is for new migrants into the UK: individuals should be tested for LTBI if they are aged 16–35 years, entered the UK from a high incidence country (≥150/100 000) or Sub Saharan Africa within the last 5 years and been previously living in that high incidence country for 6 months or longer.^20^

#### Inclusion criteria

- Age ≥16 years and ≤65 years.
- Eligible for LTBI testing with IGRA and treatment for LTBI according to UK guidance.
- Willing and able to provide written informed consent.
- Willing and able to comply with the trial, including the randomised test(s) and adherence to follow-up visits.

#### Exclusion criteria

- Allergy to C-Tb product or any of its constituents.
- Displaying any symptoms or signs of active TB disease.
- Women who are breast feeding, pregnant or plan to become pregnant during the study.*
- Women of childbearing potential not using contraception.*
  *At the time of writing, safety data on C-Tb in pregnant and breastfeeding women had not been assessed by the Medicines and Healthcare products Regulatory Agency. These criteria may be revisited once data become available.

### Interventions

Participants will be randomly allocated to the intervention arm, in which they receive the C-Tb test (Serum Institute of India, India) or the usual care arm, in which IGRA is offered as the test for LTBI. The latest generation QFT IGRA or T-SPOT.TB depending on practice at sites will be offered as the standard of care test (and from now on simply referred to as IGRA).

#### Intervention arm

Participants in the intervention arm will be managed based on C-Tb test results. C-Tb test is a skin test based on recombinant ESAT-6 and CFP10 C-Tb. The test will be administered intradermally according to the Mantoux technique.^16^ Participants will be monitored in clinic for up to 30 min after C-Tb administration. Skin induration will be assessed 2–3 days after placement; induration size of 5 mm or larger is defined as a positive reaction.^21^ Treatment of LTBI will be offered to those with a positive C-Tb result following a pretreatment assessment as per usual care (see below).

An IGRA will also be performed on all participants in the C-Tb arm in line with standard practice. Phlebotomy for IGRA will precede placement of C-Tb to avoid priming and boosting of results. Clinicians will be blinded to the IGRA result when C-Tb results are evaluated and the treatment decision is made. We will implement a study-specific standard operating procedure to ensure clinicians are blinded to the IGRA result in the rare event this is available at the time of the C-Tb reading. This scenario is highly unlikely since C-Tb results are read within 2–3 days of LTBI testing and IGRA results are obtained from designated labs up to 2–4 weeks following batch testing. Therefore, participants with positive C-Tb test results will be offered treatment before IGRA test results are disclosed. Any participants with IGRA-positive results, who have not yet been offered treatment based on a positive C-Tb result (ie, tested C-Tb negative, or unknown C-Tb result), will be assessed and offered TB preventive treatment as per usual care. Participants with positive C-Tb results but negative IGRA results who have already initiated treatment for LTBI will remain on LTBI treatment.

#### Control arm

Participants in the control arm will be tested with an IGRA alone as per usual care, and managed according to IGRA result. IGRA testing will be conducted using standardised local protocols and results will be interpreted as per manufacturer’s recommendations (cut-off for positivity 0.35 IU/L for QFT and eight spots for T-SPOT.TB). Results will be obtained within the usual time frame for that particular clinic/setting and discussed with the participant. Although time frames will be setting specific, we expect this to occur at week 4 (±2 weeks). Treatment of LTBI will be prescribed to those with positive IGRA results once TB disease is ruled out as per national guidance.^19^

#### Clinical assessments in usual care

Usual care in both arms comprises clinical assessment at baseline and during follow-up. This includes a brief review of medical history and symptom check and X-ray of the chest to rule out TB disease and further evaluation if this is suspected. Laboratory assessments may include blood testing for haematology, liver and kidney function tests, C reactive protein, HbA1c and glucose, and a blood-borne virus screen will be done for pretreatment assessment of LTBI-positive tests as appropriate within routine care, and may vary slightly by site. Participants with a positive test result either on the C-Tb or IGRA will be reviewed; a detailed history and clinical assessment will be performed, including chest radiography to rule out TB disease prior to initiation of LTBI treatment. The treatment regimen will be decided by attending clinicians according to national guidelines.^5^ Follow-up of treatment and assessment of completion will be done under usual care.

### Outcomes

#### Primary outcome

The primary outcome is initiation of LTBI treatment (within a defined 24±4 week follow-up period) based on a positive result of the randomised test (ie, C-Tb in the intervention arm and IGRA in the control arm). This will be based on all participants randomised so as to capture the overall impact of C-Tb versus IGRA testing on patient and operational processes. Participants in the C-Tb arm who did not initiate treatment based on a positive C-Tb result but started later based on a positive IGRA result will be considered not to have achieved the primary outcome.

#### Secondary outcomes

- Safety: local and systemic reactions in participants randomised to the C-Tb test.
- Process outcomes related to impact on the LTBI pathway:
  1. For participants randomised to C-Tb, failure to return for C-Tb reading within 2–3 days as recommended by the manufacturer.
  2. Acceptance of LTBI treatment among participants with a positive result of the randomised test, as determined by verbal agreement.
  3. Initiation of LTBI treatment among those with a positive result of the randomised test, as determined by confirmation of LTBI treatment medications issued by pharmacy.
  4. Losses to follow-up between diagnosis with LTBI and starting LTBI treatment.
  5. Time (days) from testing to starting preventative therapy.
  6. Completion of LTBI treatment within a 24±4 week period from starting treatment.

### Sample size

A total of 1530 participants will be recruited. This will provide the study 90% power to demonstrate non-inferiority of C-Tb compared with IGRA in terms of the proportion of participants initiating LTBI treatment based on their randomised test result, at 5% one-sided significance level (table 1).^22^ This is based on a non-inferiority margin of 6% for the absolute difference between arms, and assumes 20% of participants in the IGRA arm decide to start treatment.^23^ The non-inferiority margin can be reviewed if the event rate in the control arm differs from the original assumption within the non-inferiority frontier framework approach.^24^

**Table 1:** Power calculations for the study assuming varying proportion of IGRA arm participants starting treatment*

	Proportion of participants in IGRA arm starting LTBI treatment		
	15%	20%	25%
Sample size required to achieve 90% power	1214	1524	1786
Power of study, with overall sample size=1530	95%	90%	86%

*Based on a non-inferiority margin of 6% for the absolute difference between IGRA versus C-Tb arm and 5% one-sided significance level.

IGRA, interferon-gamma release assays.

### Recruitment

Participants will be identified from primary and secondary care settings in the UK where persons eligible for IGRA LTBI testing and treatment receive care. Contacts of TB patients identified by TB services are assessed at TB clinics for LTBI. Migrants are usually identified when they first register with a general practitioner (GP) or via database held by clinical commissioning groups and/or Flag 4 data provided by NHS Digital to Public Health England.^20 25^ We will prioritise local authority areas with a high TB incidence (≥20 per 100 000 population or over) or a high TB case burden (0.5% of all cases) that have implemented a systematic LTBI testing and treatment programme. Although initial sites and recruitment will be in London, more sites outside London may be engaged following review of recruitment rates in the first study year.

### Randomisation and allocation

Participants will be randomised centrally in 1:1 allocation ratio to the two arms, using a computerised algorithm developed and maintained by the Medical Research Council Clinical Trials Unit at University College London (MRC CTU). Randomisation will be performed using minimisation with a random element of 80%, balanced over a number of clinically important factors. To randomise a participant, data will be entered into the secure online trial database by trial team members at the site who have been trained and authorised to randomise by the MRC CTU. The database will automatically check for eligibility. Only those individuals who meet all eligibility criteria will be able to be randomised.

### Blinding

This is an open-label trial. Blinding of participants and care provider to the allocation group is not relevant, since the primary objective of this trial to examine how different modes of tests (skin test versus blood-based assay) affects initiation of LTBI treatment. However, for participants in the C-Tb arm, we will not disclose IGRA results at the time C-Tb results are read and when LTBI treatment is offered.

### Data collection methods and management

For the primary outcome, we will confirm treatment initiation by ascertaining that LTBI medication has been issued by pharmacy. The date LTBI treatment issued will be recorded within the database. Data on treatment completion will be collection of patient records at site.

Injection site reactions will be recorded. Expected adverse reactions include, but are not limited to, local pruritus, haematoma and pain. Systemic adverse events will be recorded as well as minor symptoms such as headache or nasopharyngitis, as reported in previous trials of C-Tb.^21^ A standard checklist will be used by clinicians to prompt participants and ensure adverse events are recorded. In the case of adverse events deemed serious by the attending clinician, additional tests may be done based on clinical grounds.

Information on LTBI pathway process outcomes for participants are collected during follow-up. For those who fail to attend any of the scheduled visits/appointments, attempts to contact participants will be made in line with local practice.

The MRC CTU will be responsible for overall data management and trial management. However, each site will have local responsibility for data entry into the web-based trial database.

We will follow the principles of the UK Data Protection Act to protect the personal data.

### Statistical methods

#### Primary analysis

In analysis of the primary outcome, the proportion of participants initiating LTBI treatment within the 24±4 week study follow-up period based on a positive result for their randomised test will be compared between arms. The primary analysis will be based on intention to treat approach. The difference in the proportion in IGRA versus C-Tb arm will be estimated on the absolute scale using regression models, adjusting for stratification factors. Non-inferiority will be assessed using the upper bound of the 90% CI, corresponding to 5% one-sided significance level. If the upper bound of the CI is less than 6% (the margin of non-inferiority), the C-Tb arm will be considered to be non-inferior to the standard-of-care arm. Of note, if C-Tb is shown to be non-inferior compared with IGRA, it will then be assessed for superiority.

Initiation of LTBI treatment is chosen for the primary outcome since it represents the first step in the pathway of LTBI treatment. The outcome thus captures factors directly related to test performance as well as indirect factors related to the patient, provider or context of care that influence initiation of treatment. In particular, the primary outcome is influenced by the likelihood of a positive LTBI test as well as the likelihood that a participant with a positive test result would subsequently initiate LTBI treatment. Therefore, the interpretation of the primary outcome will need to consider other components derived in the study, including, the secondary outcomes regarding participants’ acceptance and initiation of treatment among those with positive results, concordance rate between C-Tb and IGRA, and diagnostic accuracy estimates of sensitivity and specificity (see below).

#### Diagnostic accuracy

A head-to-head diagnostic accuracy evaluation of C-Tb versus IGRA will be conducted using data from participants randomised to the C-Tb arm who receive both tests. Diagnostic accuracy estimates for C-Tb including sensitivity, specificity, positive and negative predictive values will be estimated using latent class models.^26–28^ This will initially be based on data within the RID-TB:Dx trial only, but a meta-analysis approach including other relevant studies is also planned.

### Behavioural science evaluation

Quantitative surveys consisting of validated questionnaires that have been adapted for the trial will be used to assess knowledge and beliefs about LTBI, and factors influencing LTBI testing and treatment uptake within the RID-TB:Dx trial.^29–31^ This work will also assess reasons for declining participation in the diagnostic trial, and participant acceptability and impact of the test in terms of psychosocial and behavioural outcomes. Qualitative work will explore participants’ experiences of LTBI testing and treatment in greater depth to inform the development of an intervention to improve adherence to LTBI treatment. This will use cognitive interviews developed for this substudy as part of the behavioural science work package. This work package provides essential data to understand processes and outcomes deemed important by patients.

### Health economic evaluation

This will estimate if expected changes to LTBI diagnosis and/or treatment algorithms are cost-effective from the perspective of the National Health Service, using a health economic model to synthesise data obtained within the entire RID-TB programme and evidence from other sources. We will collect information on the costs participants incur in attending appointments within this trial, to allow potential future analysis from a societal perspective.

### Safety reporting

The definitions of the EU Directive 2001/20/EC Article 2 based on the principles of Good Clinical Practice apply to this trial protocol. These definitions are given in table 2. All adverse events, whether expected or not, will be recorded in the patient’s medical notes. Adverse events will be graded using the The Division of AIDS (DAIDS) toxicity grading scale.^32^ Withdrawals from the study due to local and systemic reactions will also be recorded. The investigator must assess the causality of all serious events or reactions in relation to the trial interventions using the predefined definitions. If there is at least a possible involvement of the trial treatment, the MRC CTU clinical reviewer on behalf of the sponsor will make an assessment of the expectedness of the event. Serious adverse events need to be reported to the MRC CTU within 24 hours of the investigator becoming aware of the event from the time of randomisation to the last scheduled follow-up visit at week 4.

**Table 2:** Definitions of adverse events and reactions

Term	Definition
Adverse event (AE)	Any untoward medical occurrence in a patient or clinical trial participant to whom a medicinal product has been administered, including occurrences that are not necessarily caused by or related to that product.
Adverse reaction (AR)	Any untoward and unintended response to an investigational medicinal product related to any dose administered.
Unexpected adverse reaction	An adverse reaction, the nature or severity of which is not consistent with the information about the medicinal product in question set out in approved Reference Safety Information for that product in the trial.
Serious adverse event (SAE) or serious adverse reaction (SAR) or suspected unexpected serious adverse reaction (SUSAR)	Any adverse event, adverse reaction or unexpected adverse reaction that: Results in death Is life-threatening* Requires hospitalisation or prolongation of existing hospitalisation† Results in persistent or significant disability or incapacity Consists of a congenital anomaly or birth defect Is another important medical condition‡

*The term life-threatening in the definition of a serious event refers to an event in which the patient is at risk of death at the time of the event; it does not refer to an event that hypothetically might cause death if it were more severe, for example, a silent myocardial infarction.

†Hospitalisation is defined as an inpatient admission, regardless of length of stay, even if the hospitalisation is a precautionary measure for continued observation. Hospitalisations for a pre-existing condition, that has not worsened or for an elective procedure do not constitute an SAE.

‡Medical judgement should be exercised in deciding whether an AE or AR is serious in other situations. The following should also be considered serious: important AEs or ARs that are not immediately life-threatening or do not result in death or hospitalisation but may jeopardise the subject or may require intervention to prevent one of the other outcomes listed in the definition above; for example, a secondary malignancy, an allergic bronchospasm requiring intensive emergency treatment, seizures or blood dyscrasias that do not result in hospitalisation or development of drug dependency.

Participants may be able to claim compensation for injury caused by their participation in the clinical trial in accordance with the insurance policy held at University College London (UCL).

### Monitoring and oversight

The MRC CTU is responsible for overall data management and trial management. An independent data monitoring committee (IDMC) will be formed. The IDMC will be the only group who sees the confidential, accumulating data by randomised arm. The IDMC will review study conduct and safety data regularly. The IDMC will be asked to advise on whether the accumulated data from the trial, together with results from other relevant trials, justifies continuing recruitment of further participants. The IDMC will make recommendations to the programme steering committee (PSC) as to whether the trial should continue in its present form.

The PSC has membership from the Treatment Management Group (TMG) plus independent members (approved by NIHR), including the chair and patient and public involvement (PPI) contributors. The role of the PSC is to provide overall supervision for the trial and provide advice through its independent Chair. The ultimate decision for the continuation of the trial lies with the PSC.

### Patient and public involvement

The trial was discussed with the charity TB Alert and two community representatives drawn from a migrant charity and a patient previously treated for LTBI. The behavioural science study will include work that evaluates the influence of patient, provider and institutional behavioural factors that influence engagement with, and journey through, the LTBI care pathway; this arose following PPI input.

A charity representative and one former patient read versions of the grant proposal and contributed suggestions on study design. At the protocol development stage, the following input was sought from TB Alert: study design, patient information sheet and consent form, patient-facing questionnaires used for behavioural studies.

A specific PPI work plan will be developed. This includes plans to seek and include inputs for: recruitment, patient/public engagement tools, provision of translated materials on LTBI and access to recruitment sites. We will obtain inputs from The RID-TB PPI advisory group consisting of members recruited via social media accounts, TB nurses, TB patient advocates, ex-patient contacts and voluntary/community organisations. We also plan to engage more PPI groups from migrant communities during the trial.

## Ethics and dissemination

### Ethics approval

Ethics approval has been obtained from the Health Research Authority (HRA) in the UK (269485). Any further substantial amendments will be submitted and approved by the main research ethics committee and HRA.

### Consent

Participants will be screened and consented at approved trial sites that are authorised by the MRC CTU to carry out the RID-TB:Dx trial. A copy of the participant information sheet will be given to potential participants who have been referred for LTBI testing (online supplemental appendix A). Written informed consent to enter into the trial and be randomised will be obtained after explanation of the aims, methods, benefits and potential hazards of the trial and before any trial-specific procedures are performed or any blood is taken for the trial (online supplemental appendix B).

10.1136/bmjopen-2021-050595.supp1 Supplementary data

10.1136/bmjopen-2021-050595.supp2 Supplementary data

Samples for future research will be stored at an accredited laboratory according to the University College London Human Tissue policy (based on the Human Tissue Authority’s guidance) following specific informed consent.

### Dissemination

We will report findings of the trial through publications in national and international conferences as well as in peer-reviewed journals. We will follow publication policies used in other clinical trials coordinated by the MRC CTU. All headline authors in any publication arising from the main study or substudies must have a made a substantive academic or project management contribution to the work that is being presented. Trial data will be available for sharing by request after the primary publication on approval by the TMG. We will also comply with REF Open Access policy and make a prepublication version of the manuscript available through the UCL Repository.

### Protocol version and date

This protocol is an abbreviated version of the original protocol V.5.0, December 2020. The start date of the trial is 8 October 2021.
