# Supplementary material for: Randomised controlled trial to evaluate the effectiveness of using the RD-1-based C-Tb skin test as a replacement for blood-based interferon-γ release assay for detection of, and initiation of preventive treatment for, tuberculosis infection: RID-TB:Dx study protocol
Source: BMJ Open. 2021 Dec 30;11(12):e050595. doi: 10.1136/bmjopen-2021-050595 (PMC8718463; doi:10.1136/bmjopen-2021-050595)
Supplement: Supplementary data [file bmjopen-2021-050595supp001.pdf]

APPENDIX A: RID-TB:DX CLINICAL TRIAL TEMPLATE PARTICIPANT INFORMATION SHEET (PIS) VERSION 4.0 16 DEC 2020

(To be presented on local headed paper)

Summary Participant Information Sheet for the RID-TB:Dx Clinical Trial

We are inviting you to take part in a study called RID-TB:Dx. This study aims to improve how latent tuberculosis (TB) infection or LTBI is identified within the NHS. If you have LTBI, it means you have been infected with the bacteria that cause tuberculosis (TB), but you are not ill and you do not have any symptoms. Treatment for LTBI can prevent TB.

Thank you for reading this information about the study. Please read the information carefully so you can decide if you are happy to take part. You can discuss it with friends and relatives if you wish. This page gives you an overview; please read the whole document for full details.

The study in brief:

The main study will find out if a new latent TB skin test, called C-Tb, can be used as an alternative to the standard IGRA blood-test to diagnose LTBI. C-Tb is conducted and results checked exactly like with the currently used skintest (results in 2-3 days) but it is as accurate as the "interferon gamma release assay" or IGRA (results in 4 weeks). We will assess how use of the test affects your care, confirm its accuracy, and assess cost compared to IGRA. We will also look at how people feel about LTBI, including how they feel about the C-Tb test. For people who are diagnosed with LTBI, we would also like to look at how they feel about their diagnosis and any treatment they may have ("behavioural substudy"). As well as this we would like to find out how much it cost you to come to the clinic for the appointments. This will allow us to see if the C-Tb test offers value for money for the NHS ("health economics" substudy). The behavioural and economics substudies are optional, include only people who agree, and will be questionnaire based (taking up to 10 minutes to complete each time).

Key points about how we do the study:

- We are studying a new skintest for diagnosing LTBI called C-Tb. We think C-Tb can be an equally accurate but less expensive test compared to the currently used IGRA blood test. We want to find out whether we can use C-Tb as an alternative to IGRA to diagnose TB infection.
- This study has 2 groups, one having the IGRA blood test and the other the C-Tb skintest. A computer will randomly allocate you a place in one of two groups, like the "toss of a coin". Women who are breastfeeding, pregnant or plan to become pregnant during the study will not be invited to participate. Women of childbearing potential will need to agree to have a pregnancy test and to use effective methods of contraception before being allowed to take part.
- Like all medical procedures, the tests used in this study can have unwanted side effects. They are usually minor, if they occur at all. The most common are itching and discomfort on the skin where the test was done.
- If your test results show you have LTBI, you will be referred for treatment to help prevent TB.
- Depending on your test group, this study will require you to visit the clinic one more time than if you were being tested in the usual way for latent TB. The visits will last no more than 60 minutes.
- If you take part in the study, we will also ask if you would like to donate a small (1 tablespoon) amount of blood to be used for future research.

What happens if I am interested in taking part?

If you are interested in learning more about the study, **please read the whole document for full details.** If you are interested in taking part, we will ask you some questions to see whether you can be tested for LTBI through the study. If not, your doctor will offer your usual care which is the blood test; the C-Tb test is only used as part of the study. However, we would like to ask you, if you agree, to complete some short anonymous questionnaires about your views on testing for latent TB. If you can take part in the study, we will provide you with more information and ask you to sign a consent form once you've considered the information. We will give you copies of this information sheet and consent form. If you agree we will also write to your GP to let them know that you have agreed to take part in this research.

If you have any questions about this study, please talk to your doctor or nurse:

Name of doctor or nurse:
Hospital Department:
Hospital:
Address:
Tel: 01234 XXX XXX

APPENDIX A: RID-TB:DX CLINICAL TRIAL TEMPLATE PARTICIPANT INFORMATION SHEET (PIS) VERSION 4.0 16 DEC 2020

(To be presented on local headed paper)

You are free to decide whether or not to take part in this research study. If you choose not to take part, this will not affect the care you get in any way.

- You can stop taking part in the study at any time, without giving a reason.
- Please ask us if there is anything that is not clear or if you would like more information

Full Participant Information Sheet for the RID TB:Dx Clinical Trial

1. What is latent TB?

If you have latent TB infection (LTBI), it means you have been infected with the bacteria that cause tuberculosis (TB), but you are not ill and you do not have any symptoms. If you then become ill with "active" TB disease, you could pass TB on to other people. TB bacteria are spread through the air, mainly by coughing. TB can be cured with a combination of different antibiotics which need to be taken for many months (at least 6 months). LTBI can be diagnosed and treated to help prevent TB disease from developing. The treatment for latent TB in England is usually 3 months and fewer drugs are given.

How is latent TB currently diagnosed?

One of two tests is currently used to diagnose latent TB. Both tests look for an immune response (your body's defence mechanism against germs) to TB, which shows whether you have been exposed to TB bacteria. One is a blood test called an IGRA (interferon-gamma release assay), and the other is a skin test known as the Mantoux test. The IGRA blood test can either be a "QuantiFERON Gold" blood test, or a "T-SPOT" blood test, depending on the standard of care offered at your clinic. The Mantoux test involves an injection under the skin of your forearm which will become raised and red if you have been exposed to TB bacteria. This reaction is checked in the clinic after 2-3 days in order to make a diagnosis. Both tests are considered safe, effects are minor and include, swelling, rash, itching, and discomfort, expected in a small area of the skin where the test was done.

What is the C-Tb test?

The C-Tb test is a new test for TB infection that is administered and checked just like the Mantoux test, but is more accurate than the Mantoux test and has been shown to be as accurate as the IGRA blood test. Because the reaction to the injection needs to be checked by a doctor or nurse, you will need to be able to come to the clinic for this test on a Monday, Tuesday, Wednesday or Friday and then be able to return 2-3 days later. The C-Tb test is considered as safe as the Mantoux test, causing similar effects on the skin where the test was done.

2. What does participation in the study involve?

There will be 2 different groups in the RID-TB:Dx study and everyone who takes part will be in one of them. These are:

1. **C-Tb group**: these people will have the C-Tb skin test (and also an IGRA blood test to ensure they receive the same standard-of-care as usual).
2. **Usual care group:** these people will have only the IGRA blood test.

To ensure the groups receiving each test are as similar as possible at the start of the study, a process called randomisation is used to allocate people to each group. This means a computer will randomly select which group you are in, "like the toss of a coin". Your doctor will offer you the test(s) according to your allocated group.

If you are a woman of childbearing potential, a urine pregnancy test will be done at screening to ensure you are not pregnant. You will need to agree to use an effective method of contraception for 4 weeks after entering the study.

How will I get my results?

After testing, you will need to return to the clinic to receive the results and discuss what they mean. If the results of either test indicate that you have latent TB, you will be referred to your usual care doctor or nurse, who will discuss treatment with you. You may also be referred for a chest X-ray to rule out active TB and further blood tests as part of usual care. You can get written information about latent TB from your GP or online at www.thetruthabouttb.org/latent-TB

What tests and checks will be done?

Screening: Women of child bearing potential will need to have a urinary pregnancy test to rule out pregnancy. This may need to be repeated if the screening checks are done on a different day to the day of the LTBI test.

Day 1: On the test day, if you have been allocated the C-Tb arm, we will administer the C-Tb test into your arm. You will be monitored in the clinic for up to 30 minutes after it has been administered – this will be alongside the other study activities being carried out. We will take 8ml (about one teaspoon) of blood for the IGRA test as usual. This includes enough blood to repeat the test if it does not work. We will also ask if you will give an additional blood sample of 13.5ml (about one tablespoon) to be stored for future research. This includes blood for

future genetic testing to improve the diagnosis of TB. If you do not want to give your permission for additional blood you can still take part in the main study.

Day 3-4: If you have the C-Tb test, you will be asked to visit the clinic 2-3 days later for the result to be reviewed. This is an extra visit for people in this group. If the test is positive, referral for further evaluation for treatment will be made on this visit; you will not wait until the results of the blood test.

Week 2-4: Your IGRA test result will be ready about 4 weeks after testing. We will give you a date to return to the clinic for results to be reviewed and discussed as usual. IGRA results that do not match C-Tb results will also be discussed; if the IGRA test is positive you will be offered treatment.

We may contact you about 6 months after your tests, to see how you are doing and talk about whether you had any treatment to prevent TB.

Each time you come to the clinic we will screen you for active TB, and also ask you about any other symptoms and illnesses that you have had. We will also ask you about any other health services that you have used since the last clinic visit.

Why am I being asked to take part?

Your doctor has recommended that you should have a LTBI test as you may be eligible for treatment for LTBI. We are conducting the study in your health authority area, it has agreed for us to invite anyone locally who would benefit from a latent TB test to take part.

What are the risks or disadvantages to taking part?

As with usual testing, there is a risk of mainly localised side effects. If you are in the C-Tb group, you will have an extra clinic visit than usual. Although we think C-Tb is as accurate as the IGRA, your doctor will also offer you the blood test at the same time to ensure you receive the same standard-of-care as you would normally.

What are the benefits to taking part?

The are no direct benefits to participating in this study. However, by taking part in this study, you might help us improve diagnosis and care for other people who may be at risk from latent TB or TB disease.

3. What are my rights and care during the study?

What if I experience side effects?

If you become concerned about any side effects during the study, please tell the study staff as soon as possible.

What if I my situation changes?

You may withdraw from the study at any time, or from particular aspects of the study. This will not affect the standard-of-care you receive. If you have particular concerns, however, we would ask that you talk to your study doctor or nurse first to see if they can help.

What if I have any serious concerns?

If you have any concerns about the way you have been approached or treated during the study, please talk to your study doctor or nurse. If you are still unhappy, or if you wish to complain, please use the normal NHS complaints process. If you are harmed by taking part in the study, or if you are harmed because of someone's negligence, then you may be able to take legal action.

What if new information becomes available during the course of the study?

Sometimes during a study, new information becomes available about the tests that are being studied. If this happens, your doctor will tell you about it and discuss with you whether you want to continue the study. If you decide to stop taking part in the study, your doctor will arrange for your care to continue outside of the study. Your doctor might also suggest that it is in your best interests to stop taking part in the study. Your doctor will explain the reasons and arrange for your care to continue outside the study.

What happens if the RID-TB:Dx study stops early?

Very occasionally a study is stopped early. If it happens, the reasons will be explained to you and your doctor will arrange for your care to continue outside of the study.

4. What happens to my information and results from the study?

How will we use information about you?

We will need to use information from you (and your medical records if you agree to this) for this research project. This information will include your:

- initials
- NHS number
- name
- date of birth

People will use this information to do the research or to check your records to make sure that the research is being done properly.

People who do not need to know who you are will not be able to see your name or contact details. Your data will have a code number instead. We will keep all information about you safe and secure. Once we have finished the study, we will keep some of the data so we can check the results. We will write our reports in a way that no-one can work out that you took part in the study.

What are your choices about how your information is used?

You can stop being part of the study at any time, without giving a reason (unless you have agreed to give reasons as part of our behavioural substudy), but we will keep information about you that we already have. If you choose to stop taking part in the study, we would like to continue collecting information about your health from central NHS records/ your hospital/ your GP. If you do not want this to happen, tell us and we will stop.

We need to manage your records in specific ways for the research to be reliable. This means that we won't be able to let you see or change the data we hold about you.

If you agree to take part in this study, you will have the option to take part in future research using the additional blood stored for future research. This includes blood for future genetic testing to improve the diagnosis of TB. Your blood will be stored with a code number, the link to your name will only be known to the researchers working on this study.

Where can you find out more about how your information is used?

You can find out more about how we use your information through the following ways:

- The Medical Research Council (MRC) Clinical Trials Unit (CTU) Website www.ctu.mrc.ac.uk/general/privacy-policy
- HRA Website https://www.hra.nhs.uk/information-about-patients/ www.hra.nhs.uk/patientdataandresearch
- by asking one of the research team

What will happen to the results of the RID-TB:Dx study?

When the study is completed, we will publish a summary of the results on the website of the MRC CTU at University College London (UCL): http://www.ctu.mrc.ac.uk. We will also publish the results in a medical journal, so that other doctors can see them. You can ask your doctor for a copy of any publication. We will also share and publish the results in forms suggested by patient advocacy groups involved with this study. Your identity and any personal details will be kept confidential. No named information about you will be published in any report of this study.

5. Study partners and contacts

Who is organising and funding the study?

This study is organised by the MRC CTU at UCL on behalf of The Whittington NHS Trust. The MRC CTU at UCL has run trials for many years. The study coordination, data collection and analysis and administration will be provided by the MRC CTU at UCL. You can find out more about us at www.ctu.mrc.ac.uk.

Your doctor is not receiving any money or other payment for asking you to be part of the study. University College London has overall responsibility for the conduct of the study. We are responsible for ensuring the study is carried out ethically and in the best interests of the study participants. A patient representative has been involved in the design of this study and in writing this information.

Who has reviewed the RID-TB:Dx study?

The study has been reviewed by scientists. It has been approved by the Research Ethics Committee ofLondon Harrow, and the National Institute of Health Research (NIHR) who are the funders of the study. It has been authorised by the Medicines and Healthcare products Regulatory Agency (MHRA), as well as by the NHS Health Research Authority (HRA) and the hospital's Research and Development Office.

Who can I contact for further information?

If you want further information about the RID-TB:Dx study, contact your study doctor or nurse (see below).

[Insert address and telephone number of study doctor and/or nurse]

More information is also available on our website [insert study website or address for study page on MRC CTU at UCL website].

Thank you for taking the time to consider taking part in this study.

BMJ Publishing Group Limited (BMJ) disclaims all liability and responsibility arising from any reliance placed on this supplemental material which has been supplied by the author(s)

(End

Rangaka MX, *et al. BMJ Open* 2021; 11:e050595. doi: 10.1136/bmjopen-2021-050595
