# Supplementary material for: Randomised controlled trial to evaluate the effectiveness of using the RD-1-based C-Tb skin test as a replacement for blood-based interferon-γ release assay for detection of, and initiation of preventive treatment for, tuberculosis infection: RID-TB:Dx study protocol
Source: BMJ Open. 2021 Dec 30;11(12):e050595. doi: 10.1136/bmjopen-2021-050595 (PMC8718463; doi:10.1136/bmjopen-2021-050595)
Supplement: Supplementary data [file bmjopen-2021-050595supp002.pdf]

# APPENDIX B: TEMPLATE INFORMED CONSENT FORM (ICF)

**(To be presented on local headed paper)**

**Version 4.0 16 Dec 2020**

| | |
|---|---|
| Centre Name & Number | |
| Patient ID Number | |
| Name of Researcher | |

| | | Initial to Agree |
|---|---|---|
| 1 | I have read and understood the information sheet for the RID-TB:Dx research study **[*Insert Info*: Date & Version]** and have been given a copy to keep. I have had the chance to ask questions about the project and discuss it with the study staff. I have received answers to all of my questions. | |
| 2 | I understand that my medical notes may be looked at by individuals from the Medical Research Council (MRC) Clinical Trials Unit (CTU), or from regulatory authorities where it is relevant to my taking part in this research. I give permission for these individuals to access my records. I understand that my confidentiality will be maintained. | |
| 3 | I understand that participation in this trial is voluntary and that I am free to withdraw from the trial at any time, without giving any reason and without my medical care or legal rights being affected. | |
| 4 | I understand that I may not benefit directly by participating in this study but that the research may help people with this condition in the future. | |
| 5 | In order to follow-up on my health status after my participation in the trial, I give permission for my personal details (such as NHS number, name and date of birth) to be used to obtain information about my health status from records held by NHS Digital, Public Health England, the National TB register, or any applicable national or NHS information system. I understand that this information may be obtained about me during the study and after (up to 10 years). | |
| | Women of child bearing potential only: | |
| 6a | I understand that I will have a urine pregnancy test at screening (and on the day of my LTBI test if this is done on a differet day to my screening checks) and that if this is positive I will not be invited to take part in the study. | |
| 6b | I agree to use an effective method of contraception (as disucssed with my clinician) for the duration of the study (i.e. up to the week 4 scheduled visit). | |
| | **Optional Items:**<br>***If you do not wish to give this permission, do not put your initials in the box – you can still take part in the study*** | |
| 7 | I agree for my GP to be informed of my participation in the research study.<br>**Yes** ☐ **No** ☐ | |
| 8 | I agree to participate in the Behavioural Sub-study and to complete the questionnaires.<br>**Yes** ☐ **No** ☐ | |
| 9 | I agree to participate in the Health Economics Sub-study and to complete the questionnaires. | |

| | | |
|---|---|---|
| | **Yes □ No □** | |
| 10 | I give permission for my left-over routine blood samples to be stored and made available for future research. I understand that these samples will be stored appropriately and I will not be identified by name. I understand that some of these projects may be approved separately and carried out by researchers other than the MRC CTU. I understand that the results of these research projects are unlikely to have any implications for me personally.<br>**Yes □ No □** | |
| 11 | I give permission for a blood sample to be taken and used for future research, including genetic testing to improve the diagnosis of TB. I understand that these projects may be approved separately and carried out by researchers other than the MRC CTU. I understand that I will not receive any personal results from these non-routine tests unless the researchers discover genetic information which has significant implications for my ongoing care, my future health or for that of my family. I understand that if this happens, my doctor will contact me.<br>**Yes □ No □** | |
| 12 | **I agree to take part in the RID-TB:Dx study.** | |

## Signature Page

| | | |
|---|---|---|
| Name of Participant (BLOCK CAPITALS) | Date (Day/month/year) | Signature (or thumbprint) |
| Name of Witness (BLOCK CAPITALS) | Date (Day/month/year) | Signature (if thumbprint used above) |
| Name of person taking consent (BLOCK CAPITALS) | Date (Day/month/year) | Signature |

IMPORTANT: Signed original to be kept in the Investigator Site File
One copy to be given to the participant
One copy to be kept with the participant's medical notes
